# Decoding finger movement in humans using synergy of EEG cortical current signals

**DOI:** 10.1038/s41598-017-09770-5

**Published:** 2017-09-12

**Authors:** Natsue Yoshimura, Hayato Tsuda, Toshihiro Kawase, Hiroyuki Kambara, Yasuharu Koike

**Affiliations:** 1 Institute of Innovative Research, Tokyo Institute of Technology, Yokohama, Japan; 2 Department of Neurophysiology, National Institute of Neuroscience, National Center of Neurology and Psychiatry, Tokyo, Japan; 3 ATR Brain Information Communication Research Laboratory Group, Kyoto, Japan; 4 Integrative Brain Imaging Center, National Center of Neurology and Psychiatry, Tokyo, Japan

## Abstract

The synchronized activity of neuronal populations across multiple distant brain areas may reflect coordinated interactions of large-scale brain networks. Currently, there is no established method to investigate the temporal transitions between these large-scale networks that would allow, for example, to decode finger movements. Here we applied a matrix factorization method employing principal component and temporal independent component analyses to identify brain activity synchronizations. In accordance with previous studies investigating “muscle synergies”, we refer to this activity as “brain activity synergy”. Using electroencephalography (EEG), we first estimated cortical current sources (CSs) and then identified brain activity synergies within the estimated CS signals. A decoding analysis for finger movement in eight directions showed that such CS synergies provided more information for dissociating between movements than EEG sensor signals, EEG synergy, or CS signals, suggesting that temporal activation patterns of the synchronizing CSs may contain information related to motor control. A quantitative analysis of features selected by the decoders further revealed temporal transitions among the primary motor area, dorsal and ventral premotor areas, pre-supplementary motor area, and supplementary motor area, which may reflect transitions in motor planning and execution. These results provide a proof of concept for brain activity synergy estimation using CSs.

## Introduction

Cortical motor areas have complex functional anatomy, and the purpose behind their multiple interactions in voluntary movement is not fully understood. Neurons in these areas have unique properties and interact with each other in the various stages of movement, from motor planning to action generation, in what is known as functional integration^1–3^. Considering the multiple parallel pathways between motor areas such as the primary motor area (M1), dorsal premotor area (PMd), ventral premotor area (PMv), and supplementary motor area (SMA), synchronizing temporal patterns by these areas may reflect their dynamic functions in voluntary movement.

In motor control research, muscle synergy analysis is performed to examine synchronization of muscle activity signals^4–6^. Typically, muscle synergies are calculated by applying matrix factorization methods to electromyography (EMG) signals^7^. The muscle synergy theory is based on the idea that our highly redundant musculoskeletal system requires a framework for reducing the degrees of freedom in motor control to realize complex movements^8^. The utility of muscle synergy analysis is particularly evident in studies on motor impairment. Atypical muscle synergy patterns have been examined in stroke survivors with motor impairments, suggesting that muscle synergies reflect a characteristic of motor function^9–11^. The hypothesis has been further supported through successful classification of arm and leg motion using muscle synergy^12–14^. Recent studies on humans and non-human primates also suggest the existence of brain areas that regulate muscular and kinematic synergies^15–17^. Therefore, synchronizing temporal patterns in the brain (i.e., synergy) may also possess ample information for decoding different motions. However, no approach to date has allowed for reliable classification of different motions from synergy in the brain derived from non-invasively recorded brain electric activity, e.g. electroencephalography (EEG).

In this study, we examined synchronization of brain activity signals in humans, hereinafter referred to as “brain activity synergy”. Invasive recording methods are typically effective for examining such neuronal activities. However, to provide a method with greater applicability in rehabilitation and motor learning, we chose to utilize EEG. Its high temporal resolution allowed us to capture temporal transitions in motor control. We also compensated for its low spatial resolution by applying EEG cortical current source estimation, which we previously used to reconstruct human muscle activity signals from EEG signals^18^. In this method, spatial resolution of EEG signals is computationally increased by distributing current sources (CSs) equidistantly over the cortical surface at a spacing of 2–3 mm and estimating their time series using a variational Bayesian method^19^.

Here we examined whether synchronization of distant CSs (CS synergy) reflects useable information on motor control. We applied a matrix factorization method^20, 21^ to CS signals to extract sets of CS synergy. We then attempted to decode finger-motion from the CS synergy signals using a sparse logistic regression (SLR)^22^. We compared the decoding performance with that using EEG signals, CS signals, and similarly calculated EEG synergy. We also compared temporal transitions of motor areas contributive to decoding using CS signals and CS synergy.

## Results

### CS synergy weight distributions and temporal patterns

**Figure 1:**
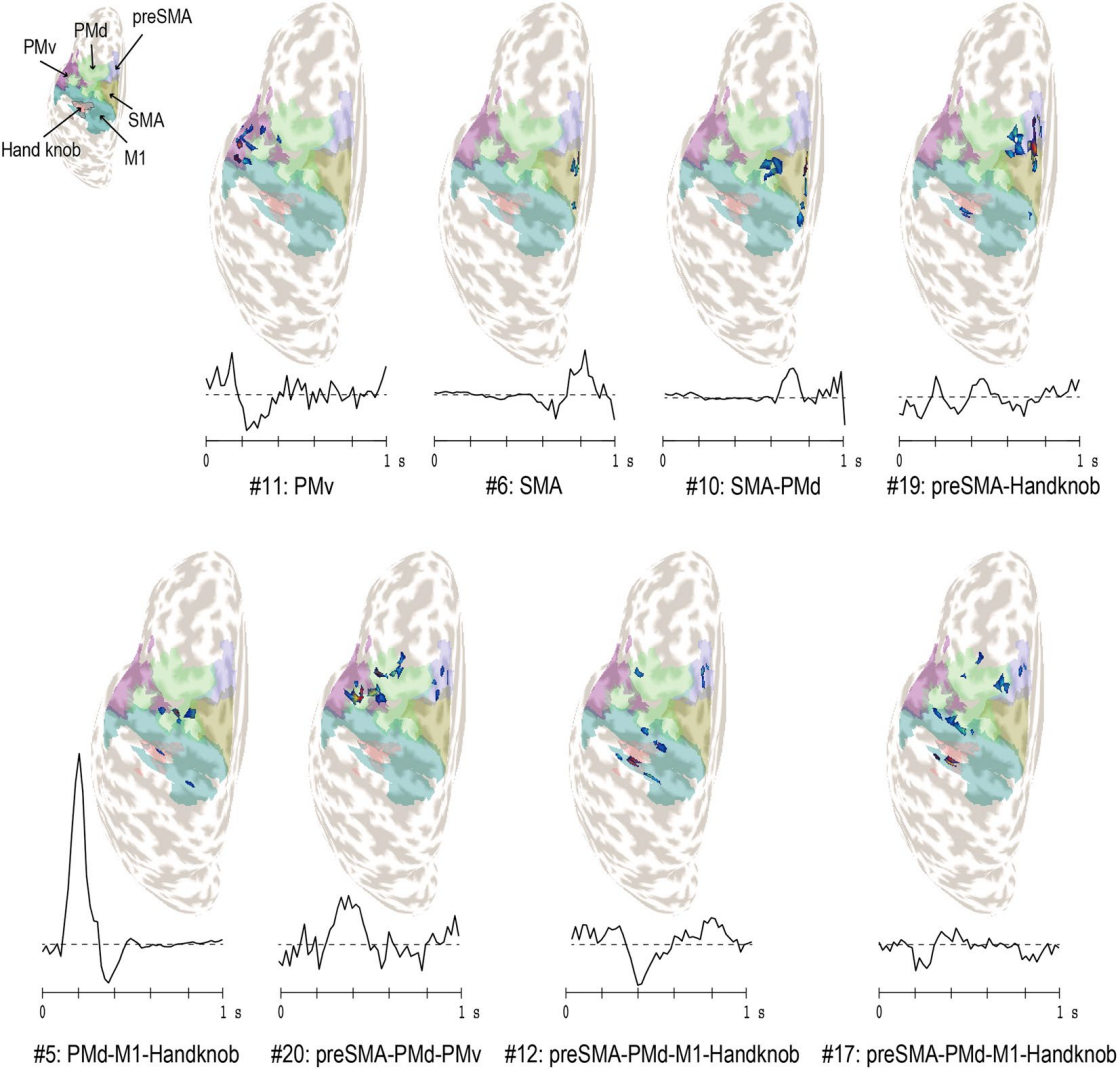
Examples of synergy weight patterns on cortical maps and temporal patterns in participant A. The topological maps are for the left hemisphere, and white and gray areas represent gyri and sulci, respectively. The six colored areas denote motor-related regions of interest: hand knob, M1, PMd, PMv, SMA, and preSMA. The weight patterns are shown as distributions of CSs. Red CSs were assigned high absolute weight values, while dark blue CSs received low weight values. The temporal patterns under the maps represent means over trials and tasks.

We recorded EEG and EMG signals during finger movement in 8 directions and estimated CS signals from the EEG signals. We then applied matrix factorization to the CS signals using principal component analysis followed by temporal independent component analysis (PCAICA)^20, 21, 23^. Figure 1 shows characteristic synergy patterns from participant A. Among 128 estimated synergies, some synergies showed synchronized CSs concentrated in specific motor-related areas, such as the PMv (synergy #11) and SMA (synergy #6). Our results also showed synchronization of CSs at distant areas. These synergies were distributed over multiple, distant motor-related areas, including the preSMA, PMd, M1, and hand knob (synergy #17), and preSMA, PMd, and PMv, (synergy #20). Overall, synergy temporal patterns varied, with positive or negative peaks tending to occur near EMG onset (around 200 ms) and initial cursor movement (around 400 ms). When we classified each synergy according to region of interest (ROI) with the highest mean synergy weight value (Table 1; see Materials and Method, Brain activity synergy estimation for details), all participants except D showed hand knob and PMv-dominant synergies.

**Table 1:** Number of synergies whose highest mean weight value was located in a region of interest.

Brain area	Participant					
	A	B	C	D	E	F
Hand knob	27	31	27	11	28	36
M1	16	13	8	28	15	14
PMd	12	17	17	15	19	15
PMv	39	28	32	31	27	23
preSMA	18	18	26	24	21	19
SMA	16	21	18	18	18	21

### Comparison of decoding performances using EEG, EEG synergy, CS signals, and CS synergy

**Figure 2:**
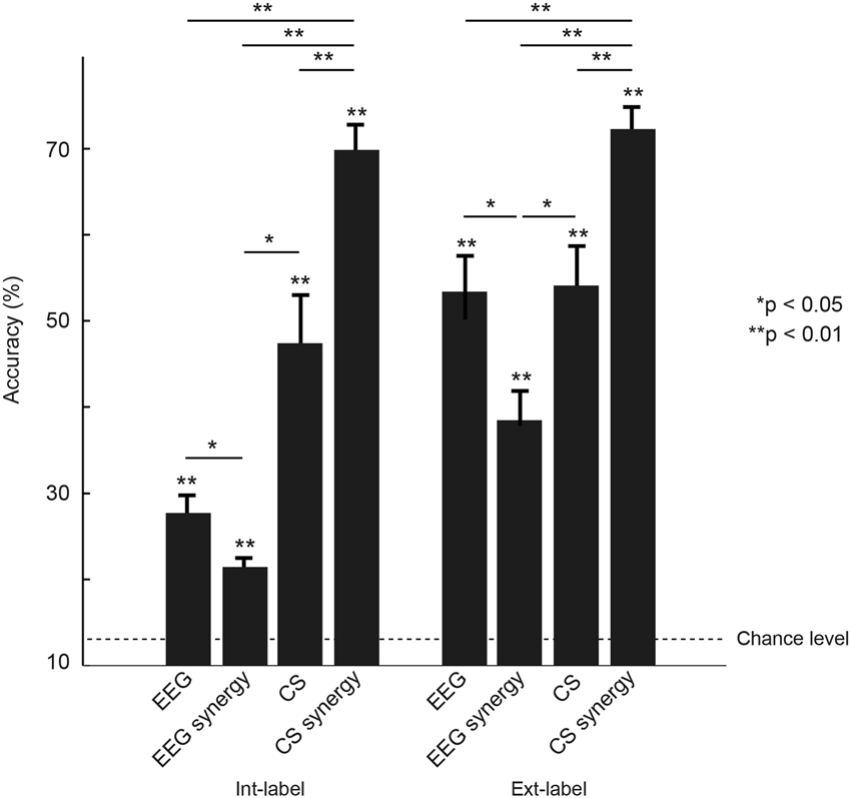
Comparisons of decoding performances using EEG signals, EEG synergy, CS signals, and CS synergy. Depicted accuracies are the mean of 80 cross-validation results for each decoding. Error bars denote standard error. Statistical differences were calculated using non-parametric permutation tests with Benjamini & Hochberg false discovery rate correction for multiple comparisons.

**Figure 3:**
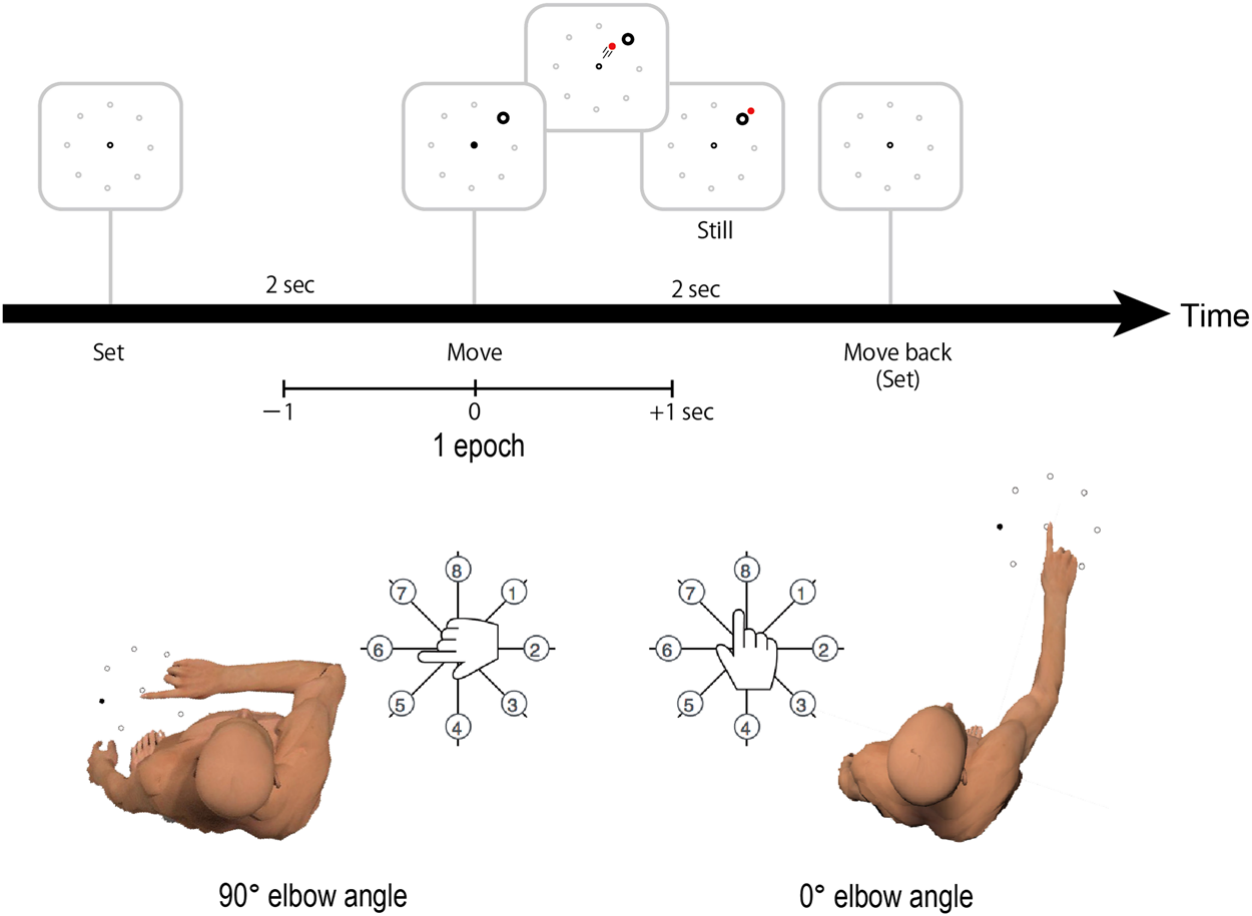
Experimental design and the two elbow angles used. Eight targets were distributed 45° apart on a circle with a 10-cm radius. A trial started when one of the targets appeared onscreen. Participants moved the cursor from the center origin to the target using a single index-finger movement on a touchpad. They maintained the position for 2 s until the target disappeared. They then moved their index fingers back to the center origin and waited for the next trial 2 s later. To discriminate between usage of intrinsic and extrinsic information, participants performed the tasks using the same target layout, but at different elbow angles 0° and 90°. Part of the illustration was created using Poser 11 Pro, (http://my.smithmicro.com/poser-3d-animation-software.html).

We compared mean decoding performances for four types of brain activity signals, EEG, EEG synergy, CS signals, and CS synergy (Fig. 2). We asked participants to perform the 8-direction finger movement tasks in two different elbow angles, 0° and 90° (Fig. 3). This allowed us to design two types of decoders, one for target direction from the center origin (right, left, etc.) and the other for finger movement (flexion, extension, etc.). In our previous work, we designed decoders for classifying motor activity during different postures according to their relation to extrinsic or intrinsic coordinate frames^24^. The extrinsic coordinate frame refers to movement with respect to the external environment, while the intrinsic coordinate frame refers to action with respect to the body. In the current study, the decoder for target direction (Ext-label) extracted extrinsic coordinate information, while the decoder for finger movement (Int-label) extracted intrinsic coordination information. We compared mean accuracies for all four signal types in each decoder using non-parametric permutation tests and Benjamini & Hochberg false discovery rate correction for multiple comparisons^25, 26^. For both labeled decodings, CS synergy showed significantly higher performance than the other signal types (Int-label, CS synergy vs. CS signals, *p* = .005, CS synergy vs. EEG synergy, *p* = .005, CS synergy vs. EEG, *p* = .005; Ext-label, CS synergy vs. CS signals, *p* = .005, CS synergy vs. EEG synergy, *p* = .005, CS synergy vs. EEG, *p* = .005). Some comparisons between the other signal types also showed significant differences (Int-label, CS signals vs. EEG synergy, *p* = .04, EEG vs. EEG synergy, *p* = .04; Ext-label, CS signals vs. EEG synergy, p = .04, EEG vs. EEG synergy, *p* = .04). These results suggest that CS synergies include necessary pattern information for 8-class decoding not only in target direction (Ext-label, 72%) but also in finger motion (Int-label, 70%). Information on target direction seemed to be extractable even from EEG signals (mean accuracy of 53%), but finger motion information could be extracted only from CS synergy signals. All signal types showed significantly higher accuracies than chance level (12.5% for 8 classes) for both labeled decodings (CS synergy, Int-label, *p* = .005, Ext-label, *p* = .005; CS signals, Int-label, *p* = .005, Ext-label, *p* = .005; EEG synergy, Int-label, *p* = .005, Ext-label, *p* = .005; EEG, Int-label, *p* = .005, Ext-label, *p* = .005).

### Temporal comparison between CS synergy and CS signals for areas contributive to decoding

**Figure 4:**
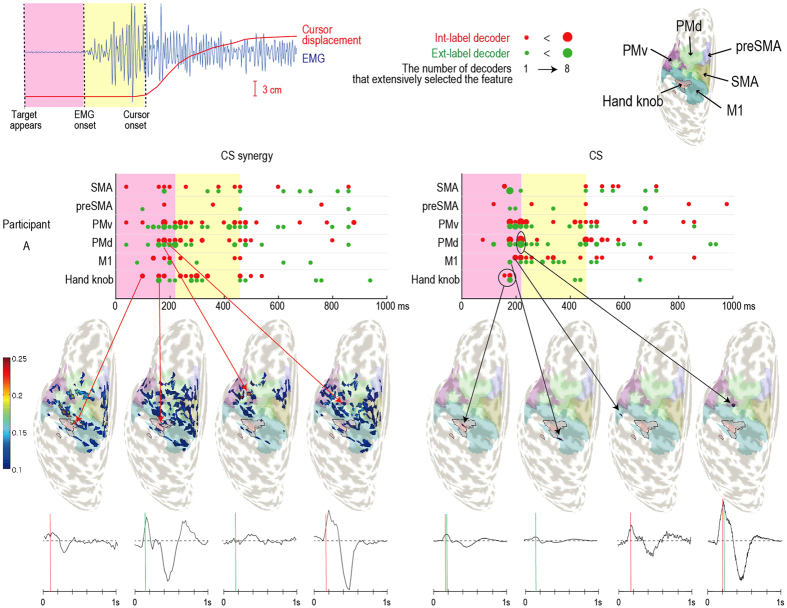
Comparisons of top 10 features selected by Int-label (red dots) and Ext-label (green dots) decoders for CS synergy (left panel) and CS signals (right panel) in participant A. Features from the 8-class decoders are plotted according to time point and area. Dot size denotes the number of decoders that selected the feature. The pink area in each graph denotes the period when the target appeared, its right border denotes average EMG onset, and the yellow areas denote the period between EMG onset and average cursor movement onset. Topological maps and their respective temporal patterns (plotted in the same manner as in Fig. 1) are provided to compare CS distributions of select CS synergies and CS signals.

Since SLR selects task-related critical features in the temporal and spatial domains, we were able to identify brain regions and timings that contributed to Int-label and Ext-label decoding. We counted the number of times each feature (synergy number or CS index) was selected by a decoder (8 classes for both Int- and Ext-label decoders) and plotted the top 10 selected features according to areas and time point (Fig. 4 for participant A; Supplementary Fig. S1 for the other participants). For both CS synergy (left panel in Figs 4 and S1) and CS signals (right panel), temporal features before cursor movement (around 450 ms) were frequently selected by all of the decoders in all participants. Although temporal activity varied across participants, tendencies were observed when comparing between CS synergy and CS signals. In CS synergy, spatial features were widely selected from the 6 motor areas, and many hand-knob-dominant synergies were selected, especially around EMG onset (border between pink and yellow areas).

Topological maps further showed that weight distribution patterns of the selected CS synergies differed between the Int- and Ext-label decoders. Specifically, a hand-knob-dominant synergy selected by Int-label decoder (red dot at 100 ms) showed weighted CSs mainly in the hand knob and M1, whereas another hand-knob-dominant synergy selected by the Ext-label decoder (green dot at 160 ms) showed many weighted CSs in PMd and SMA. Similarly, a PMd-dominant synergy selected by the Ext-label decoder (green dot at 180 ms) showed more weighted CSs in PMd than one selected by the Int-label decoder (red dot at 180 ms). In decoding with CS signals (right panel), CSs located in the hand knob were not frequently selected, and both Int- and Ext-label decoders tended to select the same CS. In addition, the top 10 selected CSs tended to cluster in a few areas (particularly PMd and PMv), most notably in participants A, B, and E.

## Discussion

In this study, we applied the matrix factorization method PCAICA to CS signals to estimate brain activity synergies. The estimated CS synergies showed topological patterns concentrated in single motor-related areas or distributed over multiple motor-related areas. Their temporal patterns representing whole activity of the synchronizing synergies tended to show positive or negative peaks at EMG onset and initial cursor movement (cursor onset). Similar to findings using muscle synergy^12–14^, efficacy of brain activity synergy was shown by decoding 8 classes of finger movements. The decoding analysis revealed that the CS synergy time-series signals provided superior decoding performance over other signal types, especially in the Int-label decoding, which requires exploiting information related to body such as joints and muscles. The temporal patterns for CS synergies seemed to reflect characteristic differences between the 8 finger movements. Our quantitative analysis further revealed the possibility to extract not only spatial patterns but also temporal transitions in brain activity for motor control. Topological maps (Fig. 4) showed that the hand knob and M1 were the main contributive areas for controlling finger movement in the intrinsic coordinate frame, while PMd and PMv were the main contributors for movement direction towards the target in the extrinsic coordinate frame, depending on their timing in transition from motor planning to execution. The differences in mapping were consistent with existing studies on non-human primates and humans^24, 27, 28^.

We had participants perform the finger movement tasks in two different elbow angles to examine the effectiveness of brain activity synergy estimation. In our previous work using functional magnetic resonance imaging (fMRI), we found human neural representations of the intrinsic and extrinsic motor coordinate frames for the wrist^24^. So here we investigated whether similar results could be observed using EEG. The high temporal resolution of EEG could potentially reveal temporal transitions between intrinsic and extrinsic coordinate frames. Although the experimental task in this study was different from that of our previous work, the concept for the representation analysis was identical. By employing the two elbow angles, 0° and 90°, we obtained data on different finger movements toward the same target (motor control in extrinsic coordinates) and data on the same finger movements toward different target positions (motor control in intrinsic coordinates). The two types of labeling for target position and finger movement and mixing the two elbow angle data allowed us to extract critical and specific information on extrinsic and intrinsic coordination. Our previous work and prior studies have shown that direction of movement is mainly encoded in PMd and PMv, but also in M1^24, 27, 29^, and muscle and joint information are encoded mostly in M1^28, 30, 31^. Although there was no significant difference in the number of times CSs in M1 and PM were selected by the Int-label and Ext-label decoders using CS synergy, the hand-knob-dominant synergy selected by the Int-label decoder at 100 ms (left-most topological map in Fig. 4) had higher CS weight values in M1 and hand knob, whereas the hand-knob-dominant synergy selected by the Ext-label decoder at 160 ms (second map from the left) had more CSs in PMd and PMv. The same tendency was found in PMd-dominant synergies (remaining two maps). The synergy selected by the Int-label decoder at 180 ms had more CSs in M1 and the hand knob than the one selected by the Ext-label decoder. Thus, CS synergy may allow for visualization of temporal transitions for intrinsic and extrinsic coordination. Moreover, even though CS signals and CS synergy shared similar contributive areas and temporal patterns, CS signals provided a significantly lower decoding performance (Fig. 2). This suggests that integrating similar CS patterns allowed SLR to select for secondary or tertiary characteristics. The variety of temporal patterns for synchronizing CSs may encode richer information relating to not only extrinsic but also intrinsic coordination. From a representational point of view, more detail in topological patterns was achieved when considering synergy and SLR weights together.

Comparing topological maps of synergies among participants, we found that all participants showed similar types of synergies. For example, some synergies included CSs in one local area of PMd, PMv, SMA, preSMA, or M1, whereas other synergies included CSs in multiple distant areas. This similarity in synergy patterns is also seen in muscle synergy analysis and reflects characteristics of normal versus impaired motor control^9–11^. Interestingly, contributive synergies for decoding differed among participants, even though hand-knob-dominant synergies tended to contribute to Int-label decoding more than Ext-label decoding.

For a more thorough investigation of CS synergy, it would be worthwhile to compare our current findings with EMG signals and muscle synergies. Since this study used simple finger-movement tasks to validate the proposed method, in future work we plan to investigate the relation between brain and muscle synergies. Furthermore, we are interested in employing statistical method such as Akaike’s information criterion to optimize the number of synergies^32, 33^. We set the number of synergies to 128 according to the matrix rank of the original EEG signals, but we found that a smaller number of synergies still showed significantly higher decoding performance than that using CS signals, EEG synergy, and EEG signals.

Applying PCA followed by temporal ICA is generally expected to provide a higher decoding performance than either method alone^34^. However, here EEG synergy (EEG plus PCAICA) showed significantly lower performance than EEG signals. Wang et al. ^34^ performed a simple binary decoding of imagery of right- and left-hand movements by applying PCAICA to EEG signals. Their method exploited activity differences between the right and left hemispheres, achieved using two EEG sensors over the central area (C3 and C4). But decoding in our study required classification of 8 different finger movements in different coordinate frames. To decode such detailed information, the spatial resolution of EEG sensors was insufficient, particularly considering the integration of synchronizing temporal patterns. Therefore, the improved decoding performance with CS synergies cannot be attributed solely to the PCAICA method, but more so to effective estimation of CS signals by the variational Bayesian multimodal encephalography (VBMEG)^19^ method we employed.

CS signals are computationally estimated from EEG signals using a machine-learning technique to solve the inverse problem. Therefore, there is always an argument against reliability. VBMEG employed in this study also requires solving an inverse problem to estimate CS signals from EEG signals, and it uses fMRI data as a hierarchical prior in Bayesian estimation. The validity of VBMEG has been shown in several studies^18, 19, 35–39^, and we have shown it to be more effective in muscle activity reconstruction than EEG signals, even when not using fMRI data. Therefore, the finding of this study, obtained without the use of fMRI, further support the validity of VBMEG.

Though the small number of participants limits the breadth of our conclusions, the decoding performance using CS synergy was significantly higher than that using other modes. Although the performance is slightly lower than that of using local field potentials in monkeys (80–90%), the 70% accuracies show potential for the method in non-invasive decoding^40, 41^. We believe the performance is a result of using a high temporal resolution method to exploit transitions in brain activity signaling during motor control. Motor-control-based brain-machine interfaces (BMIs) often employ use mu (8–12 Hz) or beta (18–26 Hz) rhythm modulations called event-related desynchronizations, occurring with motor imagery^42–44^. Yet even with their efficacy in classification, challenges still remain for online application. To realize a BMI useable in daily life, hybrid BMIs with multimodal biosignal inputs have been proposed^45–47^. Our proposed method can be technically implemented online because all processes are linear calculations if we design our filters (inverse filter for CS estimation, weight matrices for PCA and temporal ICA, and decoders for task classification) in advance. In addition, even though temporal ICA requires the number of inputs to be more than the target number of sources, CS estimation can compensate for the limited number of EEG sensors by computationally increasing the number of inputs. However, similar to other EEG-based BMIs, nonstationarity of brain activity and artifacts would pose challenges in developing a robust system. Adaptive BMIs could offer a solution to address this issue^48–50^. If the pre-calculated filters do not provide sufficient decoding performance, a calibration program could be implemented to update the filters using more recent EEG data.

## Materials and Methods

### Participants

Six healthy, right-handed, human participants (2 females and 4 males), between 30 and 51 years of age (*M* = 40.67, *SD* = 7.23), participated in this study. The study protocol was approved by the ethics committee of the University of California, San Diego (Approval No. 14353) and carried out in accordance with the Declaration of Helsinki. Written informed consent was obtained from each participant before the experiment.

### Behavioral tasks and EEG data acquisition

Participants sat on a chair with their right forearm on an arm rest (350-series, Ergorest, Siilinjarvi, Finland) and their wrist on a desk. We then asked them to perform finger movements without moving the arm. They performed computer cursor movement tasks using the right index finger on a touchpad (T650, Logitech, Lausanne, Switzerland) and the arm positioned in one of two elbow angles, 0° and 90° (Fig. 3). One trial lasted 4 s. A circle was presented at the center origin for 2 s, during which participants positioned their index fingers at the center of the touchpad. Then a target circle was presented for the next 2 s at one of eight positions distributed 45° apart on a circle with a 10-cm radius. The participants were instructed to move a red circle cursor from the center origin toward the target with a single finger movement and keep the finger in that position until the target disappeared, even if the position was not near the target. After the target disappeared, participants moved their index fingers back to the center origin, and the next trial started. One run consisted of 32 trials such that all 8 target positions were presented 4 times in pseudo-randomized order. The elbow angle was changed every 10 runs and started from 0°. The participants performed 40 runs in total, resulting in 20 runs for each elbow angle. In changing the elbow angle, the same target direction cued different finger movements. For example, target 8 indicated finger extension at 0° and adduction at 90° (Fig. 3). The experimental program was created using Psychophysics Toolbox Version 3 (Psychtoolbox-3, http://psychtoolbox.org) based on MATLAB 2013b (The MathWorks, Inc., U.S.A.), and the visual stimuli were presented on a 19-inch LCD. The cursor positions were saved at a sampling rate of 30 Hz.

We acquired EEG and EMG using a Biosemi Active Two amplifier system with active sensors (Biosemi, Amsterdam, Netherlands). EEG signals were recorded from 128 positions according to Biosemi’s equiradial layout. To identify muscle activity onset, EMG sensors were placed over the right extensor indicis and flexor digitorum. Lab Streaming Layer (https://code.google.com/archive/p/labstreaminglayer/) was used to synchronize EEG, EMG, and the experimental program for signal processing. Signals were acquired at a sampling rate of 2048 Hz. Before EEG acquisition, coordinate positions of EEG sensors as well as the nasion, left pre-auricular point, and right pre-auricular point were measured using a posture functional capacity evaluation system (zebris Medical GmbH, Isny, Germany).

### Anatomical MRI acquisition

A 3D anatomical MRI image for the head was acquired at the Center for Functional MRI in the University of California, San Diego, using a General Electric (GE) Discovery MR750 3.0 T equipped with a 32-channel receiver coil. A sagittal image was acquired using a T1-weighted spoiled gradient recalled sequence (TR = 8.132 s; TE = 3.192 ms; FA = 8°; FOV = 256 × 256 mm; matrix size = 256 × 256; 172 slices; slice thickness = 1.2 mm). The sagittal image covered the whole head, including the face, specifically for use in constructing a polygon model of the cortical surface.

### EEG data preprocessing

EEG data were loaded into MATLAB using MoBILAB toolbox^51^ and exported to the EEGLAB platform^52^ (https://sccn.ucsd.edu/wiki/EEGLAB) to perform the following preprocessing: re-sampling, high-pass filtering, and eye-movement artifact removal using temporal ICA. The EEG data were resampled to 500 Hz and high-pass filtered at a cutoff frequency of 1 Hz. Among several ICA algorithms in EEGLAB, we used adaptive component analysis (AMICA) (http://sccn.ucsd.edu/~jason/amica_web.html) for the artifact removal. Using target onset as the reference point, 80 epochs per target for each elbow angle were extracted. Each epoch had a duration of 3 s, 1 s of pre-onset and 2 s of post-onset. The epochs were saved according to task as a MATLAB file for later EEG cortical current source estimation and EEG synergy estimation.

### EEG cortical current source estimation using a hierarchical Bayesian method

CSs are equidistantly distributed on the cortical surface, considering the anatomy of sulci and gyri and assuming that activity signals from CSs propagate through cerebrospinal fluid (CSF), skull, and scalp before being recorded by EEG sensors. If the signals from CSs are reasonably estimated from EEG signals, brain activity information can be dissociated into the spatial resolution of CSs. Among several methods for CS estimation, here we used the Variational Bayesian Multimodal Encephalography (VBMEG) toolbox^19^ (ATR Neural Information Analysis Laboratories, Japan; http://vbmeg.atr.jp/?lang=en). We also used it in our previous work to reconstruct muscle activity signals from CS signals^18^.

The estimation process was conducted in accordance with standard procedures described in the toolbox documentation, unless otherwise specified. Briefly, VBMEG requires anatomical T1-weighted MRI image to calculate a cortical surface model and a three-layer model, EEG time series signal data, and EEG sensor position coordinate data for each participant. Optional functional MRI data used for priors in the Bayesian framework were not used in the current study. The cortical surface model consists of xyz coordinates of the distributed CSs on the cortical surface in the MRI image, and the three-layer model consists of boundary information for CSF, the skull, and scalp. The anatomical MRI image was first processed using the Segment function in SPM8 (Wellcome Department of Cognitive Neurology, UK; http://www.fil.ion.ucl.ac.uk/spm) to perform bias correction and tissue segmentation of gray matter. A polygon model of the cortical surface was then created using FreeSurfer (Martinos Center for Biomedical Imaging, Charlestown, MA; http://surfer.nmr.mgh.harvard.edu/). The resulting files were used to create the cortical surface model and the three-layer model in the VBMEG framework. EEG sensor positions were also co-registered on the cortical surface model using positioning software supplied with VBMEG. A leadfield matrix, which calculates EEG signals from CS signals based on sulci and gyri anatomy and electrical conductivity differences between CSF, skull, and scalp, was designed from the cortical surface model, the three-layer model, and the co-registered EEG sensor position coordinates using VBMEG.

To estimate an inverse filter that calculates CS signals from EEG signals, VBMEG requires multiple parameters. We used defaults offered by VBMEG for all parameters except the following: analysis time range = −1 to 2 s; analysis time range for current variance = −0.7 to −0.5 s; time window size = 0.5 s; shift size = 0.25 s; dipole reduction ratio = 0.2. A parameter for hierarchical prior activity was set to “Uniform” since we did not acquire fMRI. We designed an inverse filter using leave-one-trial-out cross validation, and applied the remaining trial data to the inverse filter to obtain CS data for the trial. For the following analyses, CS data located in Brodmann area (BA) 4 and 6 of the left hemisphere, which include M1, premotor area (PM), and SMA, were used since the experimental tasks involved right index finger movement. This helped to reduce computational time and memory usage, since the original number of CSs mapped onto the whole cortical surface was 20,004. BA assignment for each CS was performed using a VBMEG framework that calculates Montreal Neurological Institute (MNI) coordinates via a normalization matrix obtained with the segmentation function in SPM8.

### Brain activity synergy estimation

Among several matrix factorization algorithms, we used a PCAICA method^20, 21^ for brain activity synergy estimation since it showed similar estimation performance to the most commonly used nonnegative matrix factorization (NMF) method^53^ in muscles synergy analysis. Since NMF requires non-negative values as input, we found PCAICA, which does not have that constraint, to be appropriate for brain activity signals.

Here we define the number of EEG sensors or CSs as *M*, the number of synergies as *N*, and sampled time as 1, …, *T*. By expressing brain activity at time *t* as an *M*-dimensional column vector $b(t)$, all brain activity can be expressed as *M* $\times$ *T* matrix: $B=[b(1)\,\cdots b(T)]$.

Brain activity synergies to be estimated are defined as a series of *B*-dimensional column vectors, ${w}_{1},\,\ldots ,\,{w}_{N}$. These matrices are expressed as an *M* $\times$ *N* matrix: $W=[{w}_{1}\,\cdots \,{w}_{N}]$.

Using recorded signals *B*, we estimate *W* and *C* to satisfy the following formula under the condition of defined synergy number *N*.

1 $$B\approx WC$$

In PCAICA, we first perform PCA to reduce the dimensionality of *B*. The 1st to *M*-th principal components are calculated as follows:

2 $$\begin{array}{c}{c}_{1}^{PCA}={({w}_{1}^{PCA})}^{T}B\\ \vdots \\ {c}_{B}^{PCA}={({w}_{B}^{PCA})}^{T}B\end{array},$$

where ${c}_{i}^{PCA},\,{w}_{i}^{PCA}(i=1,\cdots ,M)$ are the estimates from PCA, ${c}_{i}^{PCA}$ are *T*-dimensional row vectors, and ${w}_{i}^{PCA}$ are *M*-dimensional column vectors that form an orthonormal basis. Therefore,

3 $$B=[{w}_{1}^{PCA}\,\cdots \,{w}_{B}^{PCA}][\begin{array}{c}{c}_{1}^{PCA}\\ \vdots \\ {c}_{B}^{PCA}\end{array}].$$

If the first *N* principal components sufficiently explain the original signals *B*, then *B* can be expressed as $B\approx {W}^{PCA}{C}^{PCA}$, where

4 $${W}^{PCA}=[{w}_{1}^{PCA}\,\cdots \,{w}_{N}^{PCA}],\,{\rm{and}}$$

5 $${C}^{PCA}=[\begin{array}{c}{c}_{1}^{PCA}\\ \vdots \\ {c}_{N}^{PCA}\end{array}]$$

Next, ICA was applied to the ${C}^{PCA}$ under condition of *N* independent components, resulting in the following formula:

6 $${C}^{PCA}=[{w}_{1}^{ICA}\,\cdots \,{w}_{N}^{ICA}][\begin{array}{c}{c}_{1}^{ICA}\\ \vdots \\ {c}_{N}^{ICA}\end{array}]={W}^{ICA}{C}^{ICA},$$

where ${c}_{i}^{ICA},\,{w}_{i}^{ICA}(i=1,\cdots ,N)$ are the estimates from ICA, ${c}_{i}^{ICA}$ are *T*-dimensional row vectors, and ${w}_{i}^{ICA}$ are *N*-dimensional column vectors. For ICA, we used the fastICA algorithm (http://research.ics.aalto.fi.ica/fastica), as applied in an existing study on muscle synergy^21^.

Finally, the original signals *B* can be expressed as

7 $$B\approx {W}^{PCA}{C}^{PCA}={W}^{PCA}{W}^{ICA}{C}^{ICA}.$$

In the brain activity synergy estimation, resulting estimates are $W={W}^{PCA}{W}^{ICA}\,$and $C={C}^{ICA}$.

We applied the PCAICA method for the EEG signals and the CS signals to compare decoding performance and topology maps from decoder weights. Synergy data were down-sampled to 50 Hz and data from 0 to 1 s (50 time points) were used for analysis. Half of the 80-trial data were used to estimate $W$ and $C$, and the remaining 40 trial data were applied to $W$ to obtain ${C}^{dec}$ in the task decoding. The number of synergies was set to 128 for the CSs, even though the number of estimated CSs in BA4 and BA6 was more than 128, considering the matrix rank of the original EEG sensor data.

### Task decoding using EEG signals, EEG synergy, CS signals, and CS synergy

We utilized two types of labeling for 8-class decoding to classify 8 finger motions (intrinsic coordination labeling (Int-label)) and 8 target directions (extrinsic coordination labeling (Ext-label)). For example, in training the Ext-label decoder, target 2 was assigned the same label for both 0° and 90° elbow angles, whereas in training the Int-label decoder, target 2 was assigned different labels for 0° and 90° since it required index finger adduction and flexion, respectively. Using the ${C}^{dec}$ from 40 trials for each task, the 8-class decoders were trained based on SLR with a Laplace approximation of a one-versus-rest algorithm (SLR-LAP-1vsR) using SLR toolbox version 1.2.1 alpha (Advanced Telecommunications Research Institute International, Japan; http://www.cns.atr.jp/~oyamashi/SLR_WEB.html)^22^. SLR identifies a set of critical features (i.e., a decoder) that maximizes decoding performance using feature selection and weight value calculation. Specifically, in SLR-LAP-1vsR, a decoder was trained for each label class, and the class with the highest probability among the 8 decoders was selected as the estimation for a test trial. Decoder weight matrices were determined through leave-one-trial-out cross validation. Therefore, one trial from each task (8-trial data) was used as test data, and the remaining trial data from each task were used for decoder training (39 trials × 8 tasks). This was repeated 40 times using unique permutations of training and testing data.

The same procedure was applied for EEG signals, EEG synergy, CS signals, and CS synergy data, respectively. The number of dimensions differed according to the number of EEG sensors and CSs, as well as the number of estimated synergies from the EEG sensors and CSs. For each signal type, decoding accuracies were calculated for Ext-label and Int-label. Mean decoding accuracies were calculated using leave-one-trial-out cross validation for each participant, and then statistical comparisons of mean accuracies were performed using non-parametric permutation tests^25^. Comparison was repeated 10,000 times using pseudo-randomized labels. Therefore, the labeling with the highest overall difference would have a *p*-value of 1/10,000, or 1.00e-04. Correction for multiple comparisons was then performed using the Benjamini & Hochberg false discovery rate method^26^.

### Quantitative analysis of selected features for Int-label and Ext-label decoders

For decoding using CS synergy and CS signals, we calculated the number of features (synergy number or CS index) selected by the 8-class decoders in Int-label and Ext-label decoding for cross-validation results higher than 40% accuracy (i.e., over three-times higher than chance level). After determining the top 10 selected features for each 8-class decoder, we plotted the number of times the each of the features was selected according to time and respective area.

We compared topological maps for the Int-label and Ext-label decoders to determine if they used signals from areas physiologically relevant to the 8 intrinsic coordinate movements and 8 extrinsic coordinate movements for each motor control phase, motor planning and motor execution. Since we previously demonstrated neural representation of the two motor coordinate frames using fMRI^24^, and the results are consistent with existing findings using non-human primates^27–29, 54, 55^, the current study also targeted the same 5 ROIs: M1, PMd, PMv, SMA, and pre-SMA. The 5 ROIs were defined based on the Human Motor Area Template (HMAT)^56^. Besides the 5 ROIs, we also added the hand knob area due to its relevance to finger movement^57, 58^. The MNI coordinates of the hand knob area were defined as x, y, z = −34 ± 4, −25 ± 3, 57 ± 11^59^. We registered the 6 ROIs onto the left hemisphere of the individual cortical brain model using an inverse-normalization transformation matrix from an MNI template to each individual participant’s native brain space. The areas were mapped onto the brain model as a part of the background in different colors. We also classified our synergies according to ROI with the highest mean synergy weight value $W$. Mean weight was calculated using synergy weight values of CSs located in each of the 6 ROIs, and the ROI with the highest mean weight was assigned as the synergy’s *dominant area*. Dominant area was determined for all synergies, and the number of synergies for each ROI was summarized in Table 1.

## Electronic supplementary material

Supplementary Information
