# Supplementary material for: Decoding finger movement in humans using synergy of EEG cortical current signals
Source: Sci Rep. 2017 Sep 12;7:11382. doi: 10.1038/s41598-017-09770-5 (PMC5595824; doi:10.1038/s41598-017-09770-5)
Supplement: Supplementary file 1 — Supplementary Information [file 41598_2017_9770_MOESM1_ESM.pdf]

Supplementary Information

Decoding finger movement in humans using synergy of EEG cortical current signals

Natsue Yoshimura[¶*], Hayato Tsuda[¶], Toshihiro Kawase, Hiroyuki Kambara, Yasuharu Koike

Supplementary Figure S1

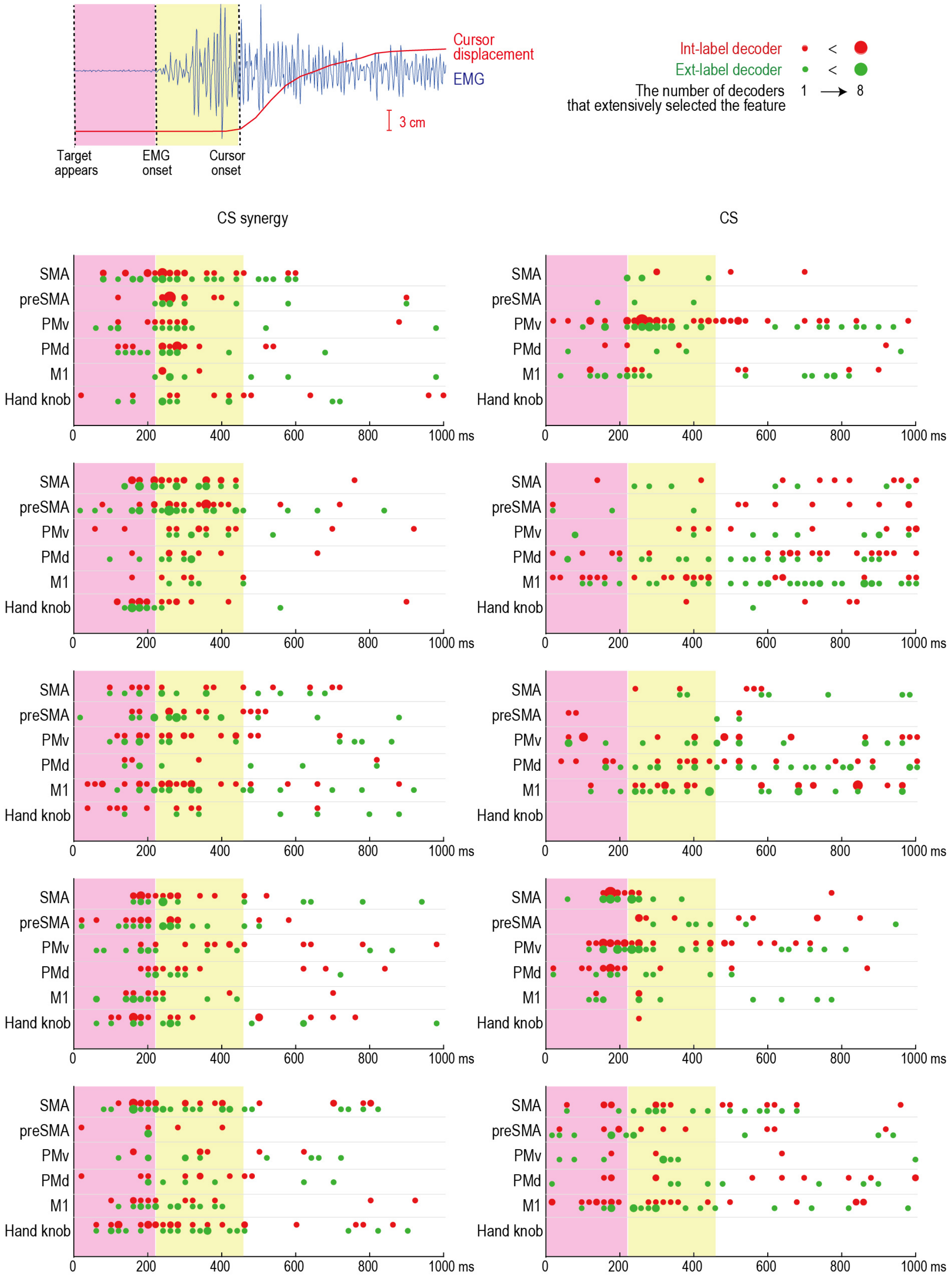

Fig. S1. Comparisons of top 10 selected features selected by Int-label (red dots) and Ext-label (green dots) decoders for CS synergy (left panel) and CS signals (right panel) in participants B-F (plotted in the same manner as in Fig. 4).
